# Novel Synthetic Polymer-Based 3D Contraction Assay: A Versatile Preclinical Research Platform for Fibrosis

**DOI:** 10.1021/acsami.2c02549

**Published:** 2022-04-25

**Authors:** Jyoti Kumari, Frank A. D. T. G. Wagener, Paul H. J. Kouwer

**Affiliations:** † Institute for Molecules and Materials, Radboud University, Heyendaalseweg 135, 6525 AJ Nijmegen, The Netherlands; ‡ Department of Dentistry - Orthodontics and Craniofacial Biology, Radboud University Medical Centre, 6525 EX Nijmegen, The Netherlands

**Keywords:** fibrosis, hydrogels, polyisocyanides, contraction models, myofibroblasts

## Abstract

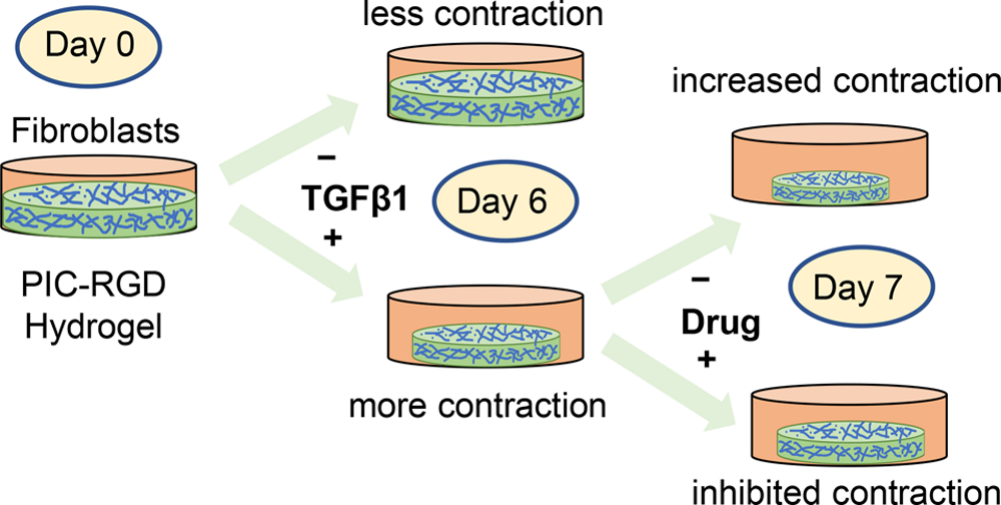

The driving factors causing fibrosis and scar formation include fibroblast differentiation into myofibroblasts and hampered myofibroblast apoptosis, which ultimately results in collagen accumulation and tissue contraction. Currently, only very few drugs are available for fibrosis treatment, and there is an urgent demand for new pharmaceutical products. High-throughput *in vitro* fibrosis models are necessary to develop such drugs. In this study, we developed such a novel model based on synthetic polyisocyanide (PIC-RGD) hydrogels. The model not only measures contraction but also allows for subsequent molecular and cellular analysis. Fibroblasts were seeded in small (10 μL) PIC-RGD gels in the absence or presence of TGFβ1, the latter to induce myofibroblast differentiation. The contraction model clearly differentiates fibroblasts and myofibroblasts. Besides a stronger contraction, we also observed α-smooth muscle actin (αSMA) production and higher collagen deposition for the latter. The results were supported by mRNA expression experiments of *αSMA*, *Col1α1*, *P53*, and *Ki67*. As proof of principle, the effects of FDA-approved antifibrotic drugs nintedanib and pirfenidone were tested in our newly developed fibrosis model. Both drugs clearly reduce myofibroblast-induced contraction. Moreover, both drugs significantly decrease myofibroblast viability. Our low-volume synthetic PIC-RGD hydrogel platform is an attractive tool for high-throughput *in vitro* antifibrotic drug screening.

## Introduction

Fibrosis is characterized by the overgrowth, hardening, and scarring of tissues and leads to functional impairment, morbidity, and mortality.^1^ It can damage the important organs of the human body including the liver, lung, kidney, and skin. Although fibrosis is considered to contribute to 30–45% of deaths worldwide, only a few therapeutics are available.^2−4^ Key mediators of fibrosis are myofibroblasts, differentiated fibroblasts that produce and deposit an excess of extracellular matrix (ECM) components including collagen. Fibrosis occurs when the production of new collagen by myofibroblasts exceeds its degradation rate. In addition, myofibroblasts induce tissue contraction, which is driven by the interaction between α smooth muscle actin (αSMA) and myosin.

As myofibroblasts play a central role in organ fibrosis, drugs targeting fibroblast differentiation or myofibroblast survival display great therapeutic potential.^5^ Currently, various drugs have been developed to treat fibrosis, but only a few of them have reached the clinical level. One of the examples is nintedanib that has been approved by the FDA for its antifibrotic activity against primary lung fibroblasts from patients suffering from idiopathic pulmonary fibrosis (IPF) and in dermal fibroblasts for patients with systemic sclerosis.^6,7^ As a tyrosine kinase inhibitor, nintedanib treats fibrosis by inhibiting activity of the vascular endothelial growth factor receptor (VEGFR), fibroblast growth factor receptors (FGFR) 1 and 2, and platelet-derived growth factor (PDGF) receptors α and β23, thereby reducing proliferation, migration, and differentiation of fibroblasts.^7^ Another promising drug is pirfenidone, an FDA-approved drug for IPF treatment^8^ that has been shown to inhibit differentiation of fibroblasts into myofibroblasts in a mouse model and to mitigate the effect of differentiated myofibroblasts.^9^ The antifibrotic effect of pirfenidone is accomplished mainly by reducing the levels of transforming growth factor (TGFβ1), basic fibroblast growth factor (bFGF), and PDGF.^10−12^

Potential drugs for treating fibrosis (or any other disease) need to undergo a severe drug screening process, including preclinical drug testing involving both *in vitro* and *in vivo* studies as well as toxicity tests. The first steps in the pharmacological analysis comprise *in vitro* studies, requiring robust biologically relevant models. For fibrosis research, a well-established test is a contraction assay that measures cell-induced macroscopic contraction of a three-dimensional (3D) gel with encapsulated (myo)fibroblasts. Currently, the gold standard matrix in these assays is collagen, which is also abundantly present in many tissues. Although functional, the collagen assays present limited opportunities for tailoring the matrix, and to avoid indiscriminate contraction of the material, the volume of the assay needs to be increased,^13^ which interferes with fast and high-throughput screening protocols. These disadvantages and the inherent variability of animal-derived materials prompted us to pursue better and more reliable assay materials.

Recently, we developed a synthetic temperature responsive hydrogel that mimics the architecture and mechanical properties of natural ECM proteins such as collagen and fibrin.^14,15^ The gels are based on polyisocyanides (PICs), and their synthetic nature allows full control over the gel structure, mechanical properties, and (bio)functionalization. Moreover, the gel is thermoresponsive, which means that a PIC polymer solution is liquid at lower temperatures (<20 °C), while as the temperature increases, the polymer chains bundle together and form a hydrogel that efficiently entraps water, even at low polymer concentrations. Like other gels built up of from semiflexible fibers (like collagen and fibrin), PIC gels do not swell.^16^ Moreover, PIC gels share unique mechanical features with collagen and fibrin gel, including strain stiffening and compression softening,^17^ underscoring the potential of PIC gels to fully capture the ECM properties in a completely synthetic environment. In contrast to the biological gel, the synthetic PIC gels are not sensitive to proteolytic cleavage, which means that remodeling can only occur using physical mechanisms.^18,19^ To induce the required cell-matrix interactions, PIC gels need decoration with a cell adhesive peptide, for example, arginine-glycine-aspartic acid (RGD)-based peptides that are commonly used to biofunctionalize artificial matrices.

In the present study, we used RGD-decorated PIC hydrogel (PIC-RGD) to develop a contraction-based fibrosis model. First, fibroblasts were cultured in a three-dimensional (3D) PIC-RGD matrix in the absence or presence of TGFβ1, which is well-known for inducing differentiation of fibroblasts into myofibroblasts. We optimized the assay to a hydrogel concentration where the difference in contraction of fibroblasts and myofibroblasts is optimal. To confirm that the observed contraction is myofibroblast-induced, the assay was validated by studying cell viability, proliferation, spreading, fibroblast differentiation, and protein and gene expression. To demonstrate the potential and expediency of the assay, we screened the effect of two antifibrotic drugs, nintedanib and pirfenidone.

## Materials and Methods

### Materials

Fetal bovine serum (FBS), Dulbecco’s modified Eagle’s medium (DMEM), Trypsin-EDTA, CCK-8, DBCO-Cy3, Triton X-100, TGFβ1, 4′,6-diamidino-2-phenylindole (DAPI), and primary antibodies against human alpha smooth muscle actin (α-SMA, clone 1A4) and produced in mice were purchased from Sigma (St. Louis, MO, USA). Primary rabbit anti-human collagen type 1 (COL-1) was bought from Cedarlane Laboratories (Burlington, Canada). Phalloidin Alexa Fluor 568, Calcein Am, and TOTO-3 were purchased from Invitrogen. Alexa Fluor 488 labeled goat anti-mouse secondary antibody was obtained from Molecular Probes Life Technologies. Alexa Fluor 488 labeled goat anti-rabbit secondary antibody was from Invitrogen (Thermo Fisher Scientific, United Kingdom). CNA35-OG488 was obtained from the Department of Biomedical Engineering (TU/e Eindhoven, The Netherlands). For purified water, Milli-Q was used.

### Synthesis of the PIC-RGD Hydrogel

PIC was synthesized as reported earlier.^20,21^ Briefly, PIC was prepared through random copolymerization of two monomers: the methyl-appended isocyano-(d)-alanyl-(l)-alanyl-tri(ethylene glycol) and the corresponding azide-appended monomer, in the presence of a Ni(ClO_4_)_2_·6H_2_O catalyst. In the polymerization, 3.3 mol % azide monomer was used, and the total monomer to catalyst ratio was 1000:1. Monomers and catalysts were mixed in toluene, and the reaction mixture was stirred overnight at room temperature. The completion of the reaction was confirmed by FTIR,^21^ which showed the disappearance of the isocyanide absorption at 2140 cm^–1^. The polymer was then precipitated in diisopropyl ether under vigorous stirring and isolated by centrifugation. The polymer was further dissolved in dichloromethane and precipitated in diisopropyl ether two more times.

PIC biofunctionalization followed earlier protocols.^22^ The GRGDS peptide (H-Gly-Arg-Gly-Asp-Ser-OH, Bachem Germany, 1.4 mg) dissolved in borate buffer (0.25 mL) was mixed with DBCO-PEG4-NHS (Bioconjugate Technologies, Scottsdale, US, 2.1 mg) dissolved in DMSO (0.3 mL). The mixture was reacted at room temperature for 4 h. Full conversion of the reaction was confirmed with mass spectrometry. The azide-decorated polyisocyanide was dissolved in acetonitrile (2.5 mg/mL), the DBCO–peptide conjugate was added in a 1:1 ratio of azide to DBCO, and the reaction was stirred at room temperature for 24 h. After the reaction, the GRGDS-conjugated polymer (PIC-RGD) was precipitated in diisopropyl ether, collected by centrifugation, air-dried, and stored in the form of a pellet. The PIC-RGD pellets were then UV sterilized at 254 nm for 10 min and dissolved in sterile PBS at a concentration of 8 mg/mL at 4 °C overnight. The dissolved polymer was then aliquoted and stored at −20 °C to be used at the time of cell seeding.

### Pore Size Characterization

Qualitative pore size analysis of the PIC-RGD hydrogels using confocal microscopy followed earlier described procedures.^23^ The different concentrations of PIC-RGD solutions were mixed with DBCO-Cy3 (20 μM) and incubated at 4 °C for 30 min. After incubation, the solution was directly added on a microplate to form the hydrogel and incubated at 37 °C for 1 h, followed by imaging on a Leica SP8× confocal microscope.

### Rheology

Rheology experiments were carried out in a rheometer (Discovery HR-1, TA Instruments) using a steel parallel plate geometry (20 mm). A hydrogel solution was loaded on the precooled (5 °C) bottom plate, and after lowering the top plate, a temperature ramp to 37 °C at a rate of 8 °C/min was started, followed by a time sweep of 15 min. Mechanical data in the manuscript is recorded in the linear viscoelastic regime at 1% strain with a 1 Hz frequency.

### Cell Culture and Cell Seeding in the PIC-RGD Hydrogel

Fibroblasts derived from human foreskins (HFFs) were cultured in Dulbecco’s modified Eagle’s medium (DMEM) containing 10% fetal bovine serum (FBS) and 1% penicillin/streptomycin until 80–90% confluency as described previously.^24^ Cells were used between passages 7–12. At the time of seeding, cells were harvested using trypsin-EDTA and resuspended in the complete medium after centrifugation. Hydrogels of different polymer concentrations (1, 2, 4, and 8 mg/mL) were prepared using a polymer stock solution with a similar number of cells at a final density of 10^6^ cells/mL. Cell-polymer solutions of 100 μL or 10 μL were used in the case for 96-well plates and microplates (uncoated, Ibidi GmbH, Martinsried, Germany), respectively. After seeding, the plates were then incubated at 37 °C, 5% CO_2_ for 30 min. The medium (200 μL for 96-well plates and 50 μL for microplates) in the absence or presence of human TGFβ1 (10 ng/mL) was added on top of the hydrogels. The medium was refreshed on day 3. Cell seeding, medium addition, medium refreshment, and staining procedures were carried out on a hot plate (37 °C) inside the cell-culture hood.

Collagen gels were used as the control for the PIC-RGD hydrogels. The collagen gel solution was prepared using collagen type I, which was then mixed with α-MEM (10X), HEPES (1M), NaHCO_3_ (7.5%), and NaOH (10M) to a final concentration of 0.5 and 1 mg/mL. The number of cells and the subsequent cell seeding process were kept similar as described above.

### Cell Spreading on the PIC-RGD Hydrogel

Cell spreading was analyzed by bright field imaging and by confocal fluorescence microscopy after cytoskeleton staining using Phalloidin Alexa Fluor 568. The imaging and staining were performed at days 3 and 6. Before staining, the medium from cell-seeded hydrogels was removed, and the hydrogels were fixed for 10 min with 4% paraformaldehyde at 37 °C. The cells encapsulated in hydrogels were permeabilized using 0.5% Triton X-100/PBS. The phalloidin (400X stock solution, 1:400) was added for 1 h, followed by washing with warm 0.05% Tween-20/PBS, and counterstained with DAPI (1 mg/mL, 1:100) for 15 min. Finally, hydrogels were washed with warm PBS, and samples were imaged on a Leica SP8× confocal microscope.

### Gel Contraction Assay

Microplates were used to measure contraction in PIC-RGD hydrogels and collagen gel. At each time point, bright field images were taken using an inverted microscope using a 2.5× objective. Covered areas were measured using NIH ImageJ software version 1.51. Contraction is expressed as the percentage of hydrogel contracted compared to the total area of the plate following eq 1
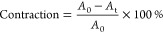
1where *A*_0_ is the area of the plate, and *A*_t_ is the area occupied by the hydrogel at time *t*.

### Live–Dead Assay

Live–dead assays were carried out in the microplate. For staining, first, the spent medium was removed from the hydrogel. Solutions of Calcein AM (2 mM; 1:1000) and TOTO-3 (1 mM; 1:1000) were prepared in the medium (37 °C) and added on the hydrogel. The plate was then incubated for 1 h at 37 °C, 5% CO_2_ followed by one single PBS wash. The images were taken using a Leica SP8× confocal microscope at 37 °C.

### Cell Viability Assay

Cell viability was analyzed in a 96-well plate using a Cell Counting Kit-8 (CCK-8) assay following the manufacturer’s instructions. Briefly, the CCK-8 solution (1:10) was prepared in the basal medium, and 100 μL of the solution was added on top of the hydrogel after removing the spent medium. After 2 h of incubation at 37 °C, the absorbance at 450 nm was measured using a plate reader.

### QuantiFluor dsDNA Assay

The DNA concentration was analyzed using a QuantiFluor dsDNA assay kit (Promega, Madison, USA). On the day of analysis, the cell culture medium was removed from the hydrogel. After washing with PBS, the 96-well plate was placed on ice for 20 min to liquify the hydrogel. Then, 500 μL of Milli-Q was added to each well, and the plate was stored at −20 °C. For analysis, the sample was further diluted with Milli-Q to reach fluorescence values within the range of the standard curve. The quantification was performed according to the manufacturer’s instructions. Briefly, the QuantiFluor dye was prepared in a 1× tris-EDTA buffer (1:200). The diluted sample (in total 50 μL) was mixed with the dye solution (50 μL), pipetted into a flat black 96-well plate, and incubated at room temperature in the dark for 5 min. In a plate reader (Tecan Spark M10 plate reader), the samples were excited at λ = 485 nm, and the fluorescence intensity was recorded at 530 nm. The reported fluorescence intensities are normalized to day 0 values.

### Total Collagen Production

Total collagen production was analyzed using a fluorescent collagen probe known as CNA35-OG488, which binds to all different types of collagen.^25^ The staining protocol of the manufacturer was used. Briefly, on day 6, the cells encapsulated in the hydrogels were fixed by adding 4% paraformaldehyde and incubating for 10 min at 37 °C. CNA35-OG488 in PBS (1 μM) was added to the hydrogel, and the samples were incubated overnight at 37 °C. The hydrogel was then washed with PBS. DAPI (1 mg/mL, 1:100) was then added for 15 min to stain the nuclei. Images were captured using a Leica SP8× confocal microscope.

### Fluorescent Immunostaining

For αSMA imaging, the cell encapsulated hydrogels were fixed with 4% paraformaldehyde at 37 °C, 5% CO_2_ for 10 min in the microplate. After PBS washing, the hydrogel was permeabilized with 0.5% Triton X-100/PBS for 20 min and blocked in 10% bovine serum albumin (BSA) for 1 h. Then, the hydrogels were incubated with primary mouse anti-human αSMA (1:800) or rabbit anti-human COL-1 (1:200) at 37 °C in an incubator overnight. After washing with warm 0.05% Tween-20/PBS, Alexa Fluor-488 labeled goat anti-mouse or goat anti-rabbit secondary antibody (1:200) was added for 1 h and incubated at 37 °C. DAPI (1 mg/mL, 1:100) was then added for 15 min to stain the nuclei. Images were captured using a Leica SP8× confocal microscope.

### RT-PCR

To extract the cells from the PIC-RGD hydrogel, the excess medium was pipetted from the culture, followed with a single wash with PBS. Then, cold PBS (4 °C) was added on the hydrogel for 5 min, and the solutions were collected into a tube to centrifuge at 400 rcf for 5 min at 4 °C. Here, cold PBS facilitates the liquification of the hydrogel, allowing easy isolation of the cell pellets. Total RNA was extracted from the cell pellets using the RNeasy Micro Kit (Qiagen) according to the manufacturer’s instructions. The RNA was then converted into cDNA using the iSCRIPT cDNA synthesis kit (BIO RAD). qRT-PCR was done using SYBR green supermix (BIO RAD) using forward and reverse primers (Table 1) with the reaction conditions of 3 min at 95 °C, followed by 39 cycles of 95 °C for 15 s and 60 °C for 30 s. The average *C*_t_ value of experimental, control, and reference (*GAPDH*) genes was used to calculate Δ*C*_t_ and ΔΔ*C*_t_ as follows:
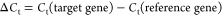
2
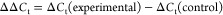
3The gene expression is represented in terms of fold change (= 2^–ΔΔ*C*_t_^).

**Table 1:** Primer Sequences for Real Time PCR

gene	forward primer	reverse primer
*GAPDH*	TGC ACC ACC AAC TGC TTA GC	GGC ATG GAC TGT GGT CAT GA
*αSMA*	GCT CAC GGA GGC ACC CCT GAA	TCC AGA GTC CAG CAC GAT G
*COL1α1*	CAG CCG CTT CAC CTA CAG C	TCA ATC ACT GTC TTG CCC CA
*p53*	GCA TTC TGG GAC AGC CAA GT	GTG GTG ACT GCT TGT AGA TG
*Ki67*	AAA CCA ACA AAG AGG AAC ACA AAT T	GTC TGG AGC GCA GGG ATA TTC

### Antifibrotic Drug Treatment

To study the effect of nintedanib and pirfenidone on the developed contraction model, fibroblasts were cultured in the PIC-RGD hydrogel in the presence or absence of TGFβ1 (10 ng/mL) until day 6. At day 6, nintedanib (1 μM) or pirfenidone (500 μg/mL) was added in separate wells in the presence or absence of TGFβ1 (10 ng/mL). After 24 h, the effects of the drugs were checked in terms of contraction, cell viability, αSMA production, and gene (*αSMA*, *Col1α1*, *p53*, and *Ki67*) analysis.

### Image Analysis

All the immunofluorescence images were analyzed using ImageJ following earlier described protocols.^26^ Briefly, cell areas, mean gray values, and integrated densities were measured for several randomly selected areas of interest as well as for background regions (areas without fluorescence). The total number of cells was counted in each region of interest to normalize the fluorescence intensities. In the manuscript, the quantified fluorescence is expressed as the corrected total cell fluorescence (CTCF), which is calculated from CTCF = integrated density – (area of selected cell × mean fluorescence of background readings).

### Statistical Analysis

All data in the manuscript were presented as mean ± standard deviation. Sample sizes are given in the caption. The significance of the differences between the mean values of the two groups is assessed by the Student’s *t* test where *P* < 0.05 was considered as statistically significant.

## Results

### Physical Properties of PIC-RGD Hydrogels

**Figure 1:**
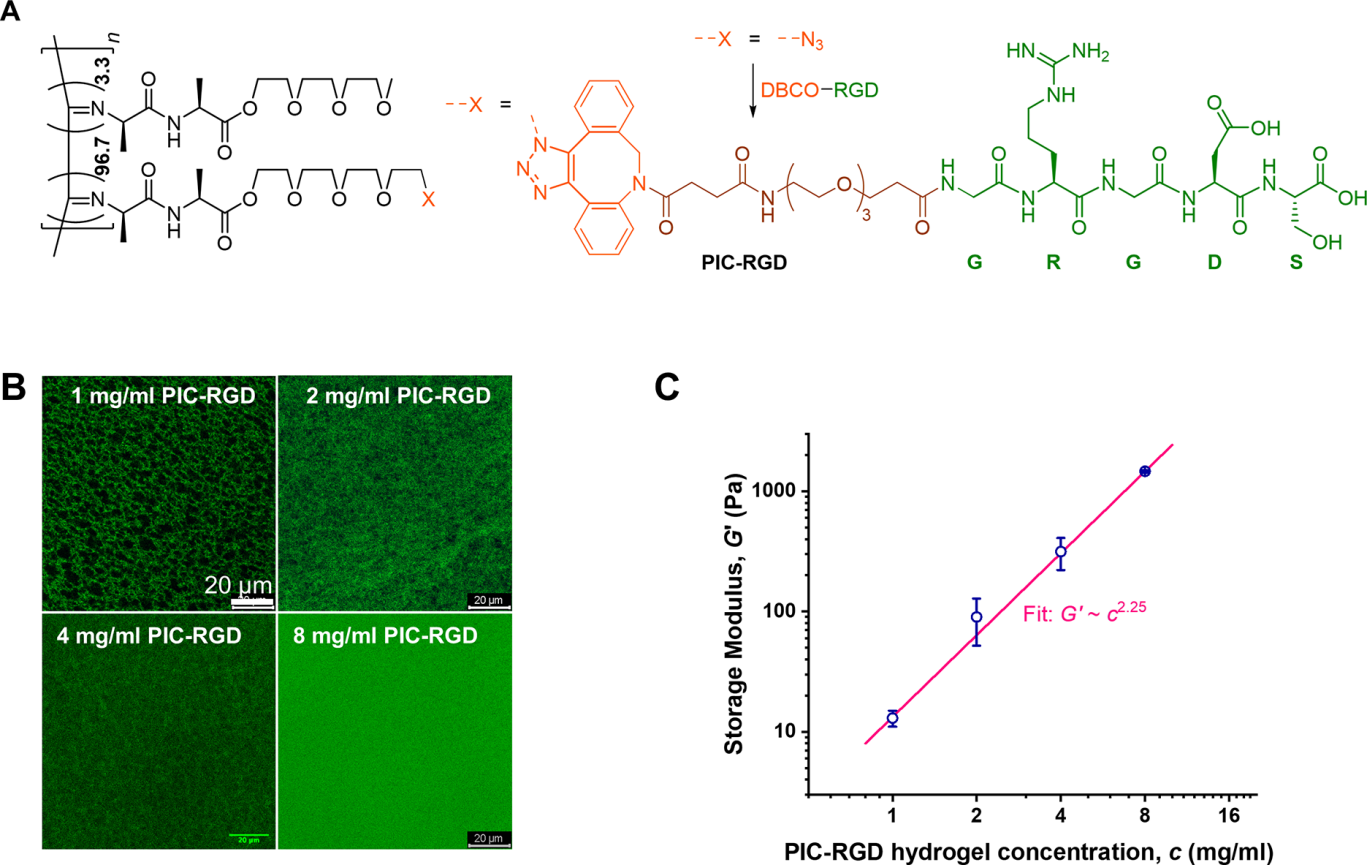
Structure and physical properties of PIC-RGD hydrogels. A) RGD conjugation reaction to form the PIC-RGD polymers. B) Confocal fluorescence microscopy images of PIC-RGD hydrogels at different concentrations. The scale bar is 20 μm. C) Storage modulus *G*′ of different PIC-RGD hydrogels. The experimental data has been fitted to a power law function.

To render the polymers biocompatible, PIC that was statistically decorated with azide groups^20^ (3.3 mol %) reacted with a DBCO-functionalized cell binding peptide (RGD) via the strain-promoted azide–alkyne click chemistry (SPAAC) reaction, as described before^27^ (Figure 1A). A detailed description is given in the Materials and Methods section. Gels were formed by stirring the solid polymer with the medium at 4 °C and after full dissolution heating the sample to 37 °C. The PIC fibers form a 3D interconnected fibrillar network leading to a heterogeneous porous architecture that forms the PIC hydrogel.^23^ Confocal images of the Cy3-labeled hydrogel, obtained after conjugating a small fraction of the azide moieties with a DBCO-functionalized Cy3 dye (Cy3-DBCO) using the SPAAC reaction, clearly show the porous morphology of the gel. For a gel with a concentration of 1 mg/mL PIC-RGD, the pore size measures approximately between 1 and 5 μm (Figure 1B), which is in line with a more detailed study on PIC gel porosity,^23^ where a quantitative analytical approach was used to determine gel pore sizes as a function of PIC concentration, which we carried out earlier. We note that, in our hands, cryoSEM experiments give beautiful images, but the results are highly susceptible to artifacts from sample preparation; and we prefer not to extract quantitative data from the images. Although for higher polymer concentrations (2, 4, and 8 mg/mL PIC-RGD), it becomes increasingly difficult to analyze the exact pore sizes through confocal microscopy, and the decrease in pore size with increasing concentrations is easily visible.

The mechanical properties of the gels at different concentrations were assessed by oscillatory shear rheology (Figure 1C). Gel formation as a function of temperature is clearly observed from the rheology traces where the gelation temperature, *T*_gel_ ∼ 20 °C, is marked as the onset of the increase in storage modulus *G*′ (Figure S1A). At *T* = 37 °C, all concentration gels formed a mostly elastic material with *G*′ ≫ *G*″ where *G*″ represents the loss modulus. Further analysis showed that over the fully probed frequency regime (0.1–10 Hz) *G*′ and *G*″ are constant (Figure S1B). Note that for the low concentrations *G*″ is too small to give accurate data. The increase of the storage modulus *G*′ with the polymer concentration follows the power law *G*′ ∼ *c*^2.25^ where *c* is the polymer concentration. This result is fully in line with the theory for these types of fibrous (semiflexible) polymer networks^28^ and with previous results.^29^

### TGFβ1 Treatment-Induced Fibroblast Differentiation into Myofibroblasts

To be able to discriminate between the behavior of fibroblasts and myofibroblasts, we confirmed that the treatment with TGFβ1 indeed activated our human foreskin fibroblasts. The cells were cultured in the presence of 10% FBS and 10 ng/mL TGFβ1 for 3 days on a tissue culture plate (Supplementary Figures S2A and S2B). The initial concentration of TGFβ1 was chosen based on a literature protocol.^24^ Immunostaining for αSMA confirmed the differentiation of fibroblasts into myofibroblasts in the presence of TGFβ1 (*P* = 0.0007). Note that lower serum concentrations reduced myofibroblast differentiation (data not shown). In the remainder of the manuscript, fibroblasts that were cultured in the presence of TGFβ1 are considered myofibroblasts.

### Cells Spreading Inside the PIC-RGD Hydrogels

**Figure 2:**
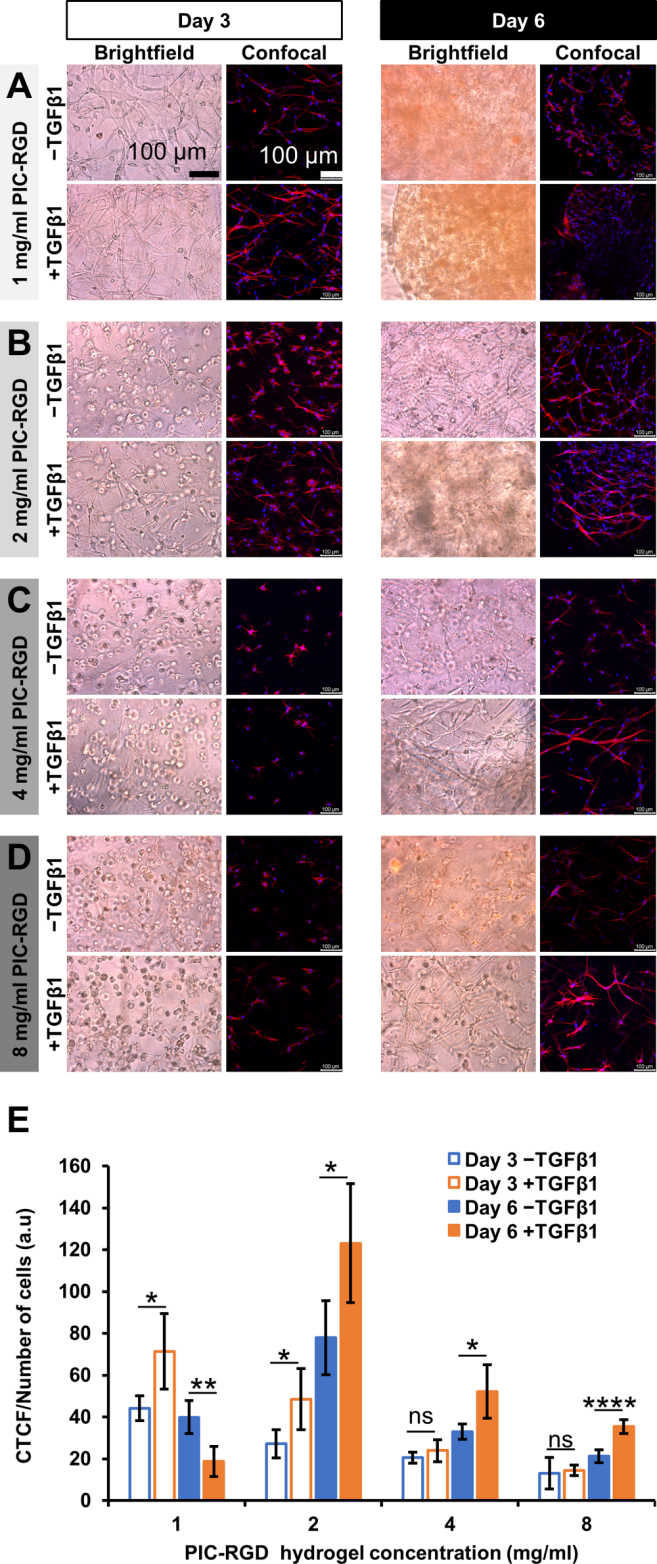
Cell spreading in different concentrations of the PIC-RGD hydrogel. A–D) Bright field and confocal images showing cell spreading in the 1, 2, 4, and 8 mg/mL PIC-RGD hydrogel at day 3 (left column) and day 6 (right column). E) Quantitative analysis of confocal images. CTCF of the red channel was calculated and normalized to the number of cells; number of images *n* = 5. Staining: F-actin; red stained with Phalloidin Alexa Fluor 568 and counterstained with DAPI for nucleus (blue) staining. Scale bars: panels A–D, 100 μm. Statistical analysis with an unpaired *t* test. *P*-values > 0.05 are considered not significant; significant differences: *, *P* ≤ 0.05; **, *P* ≤ 0.01; ***, *P* ≤ 0.001; ****, *P* ≤ 0.0001.

As fibroblast and myofibroblast cell behavior will strongly depend on the mechanical properties of the microenvironment, we seeded the cells in hydrogels of different concentrations (1, 2, 4, and 8 mg/mL) and, thus, different stiffnesses (*G*′ = 10–1460 Pa). Cell spreading at days 3 and 6 after incubation was visualized with bright field microscopy and with confocal fluorescence microscopy after staining for F-actin and nuclei (Figure 2A–D). The fluorescence data was quantified and normalized to the number of cells to give corrected total cell fluorescence (CTCF) values (Figure 2E).

At day 3, both fibroblasts and myofibroblasts spread more in lower concentration PIC-RGD than in the higher concentration gels. Comparing fibroblasts and myofibroblasts, we find that myofibroblasts spread more in the low concentration hydrogels (1 and 2 mg/mL, Figure 2A,B), and we observe no differences when the gel becomes more concentrated (Figure 2C,D). At day 6, cell spreading in the 1 mg/mL gel decreased due to breakage of the hydrogel. Moreover, this hydrogel was very fragile and contracted too much to consider further. For the 2, 4, and 8 mg/mL gels, spreading increased with respect to day 3. Comparing cells cultured in the absence and presence of TGFβ1 shows that the myofibroblasts spread more (*P* = 0.0167, 0.0124, and 0.0001, respectively). Increased cell spreading also led to more cell–cell interactions, which, subsequently, promoted hydrogel contraction. In the case of collagen, due to its highly contractive nature it is difficult to observe cell spreading (data not shown).

### Macroscopic Contraction Studies

**Figure 3:**
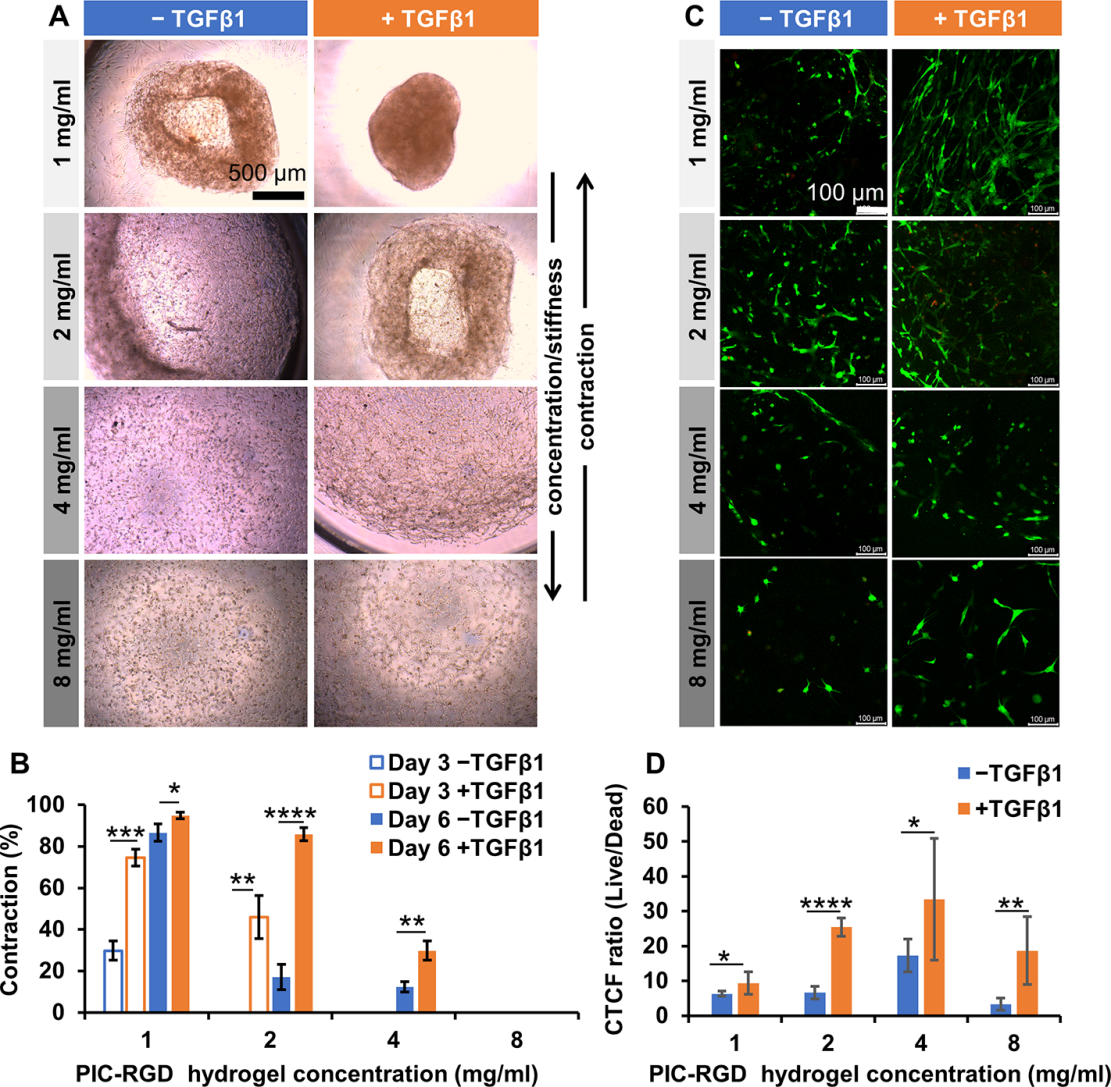
Hydrogel contraction and the live–dead assay for different concentrations of PIC-RGD hydrogels. A) Bright field images showing contraction in hydrogels seeded with fibroblasts in the absence or presence of TGFβ1 at day 6. B) Percentage contraction in the 1, 2, 4, and 8 mg/mL PIC-RGD hydrogel at day 3 and day 6 (*n* = 3). C) Confocal images of live–dead assays at day 6. Living cells and dead cells were stained with Calcein-AM (green) and TOTO-3 (red), respectively. D) Quantitative analysis of the live–dead confocal images. Data is given as the ratio of the CTCF values of the green and red channels. Number of images analyzed: *n* = 8. Scale bars: panel A, 500 μm and panel C, 100 μm. Statistical analysis with an unpaired *t* test. *P*-values > 0.05 are considered not significant; significant differences: *, *P* ≤ 0.05; **, *P* ≤ 0.01; ***, *P* ≤ 0.001; ****, *P* ≤ 0.0001.

Macroscopic hydrogel contraction was measured following fibroblast encapsulation (in the absence or presence of TGFβ1) using bright field imaging. The results are quantified as the relative decrease in the hydrogel area compared to the gel at the time of seeding (or the size of the well), see eq 1. As expected, PIC-RGD hydrogel seeded myofibroblasts displayed more contraction than those with fibroblasts (Figure 3A). In addition, the contraction strongly depended on the polymer concentration (Figure 3B). In the case of 1 mg/mL PIC-RGD, both the myofibroblast cultures (day 3: 74% ± 4%, day 6: 94% ± 2%) and the fibroblast cultures showed contraction (day 3: 30% ± 5%, day 6: 86% ± 4%), albeit the latter less than the former (Figure S3). Contrarily, at the highest PIC-RGD concentration (8 mg/mL), even after 6 days no contraction was observed in any of the gels, irrespective of the presence of TGFβ1. Interestingly, in the 2 mg/mL PIC-RGD hydrogel, fibroblasts showed no contraction at day 3, whereas the myofibroblast contracted the gel 46% ± 10%. Further at day 6, the difference was similarly large: fibroblast contraction of 17% ± 6% versus myofibroblast-induced gel contraction of 86% ± 3%. In 4 mg/mL hydrogels, the difference in contraction between fibroblasts and myofibroblasts was less pronounced: fibroblasts showed no contraction at day 3 and 12% ± 2% at day 6; myofibroblasts contracted by 30% ± 5% at day 6.

The behavior in the PIC-RGD hydrogels was compared with the commonly used collagen model, which represents the gold standard in the field. We note that in our setup, the volume of the assays (10 μL) is many times smaller than for commonly used collagen assays. As a result, our collagen results may not be completely in line with the results of “default” collagen-based assays. We observed that the 10 μL samples of 0.5 and 1 mg/mL collagen nearly completely contracted (contraction >95%) already at day 1 after encapsulation (Figures S4A and S4B). Moreover, we found no difference in contraction between the 0.5 and 1 mg/mL collagen gels.

### Live–Dead Staining of Cells Encapsulated in PIC-RGD Hydrogels

The cell culture matrix as well as its contraction may affect cell viability and proliferation. We measured viability in all concentrations of PIC-RGD hydrogels through a live–dead assay (Figure 3C) with staining live cells with calcein-AM (green) and dead cells with TOTO-3 (red). The fluorescence images show that in all cases the larger share of the cells is alive. In the case of hydrogels, it is difficult to count the viable and dead cells, and therefore, we determined the CTCF of green and red channels; the results were presented as a ratio. Subsequent image analysis showed that the ratio of CTCF of viable cells to dead cells was more than 1 in the different PIC-RGD hydrogels at day 6 (Figure 3D). In the collagen gel, however, a significant number of cells were dead, in the presence and absence of TGFβ1 (Figure S4C). Image analysis confirmed these results, showing ratios of live to dead cells < 1, both at day 3 and day 6 (Figure S4D).

Based on the high difference in contraction between the fibroblasts and the myofibroblasts, in combination with the positive live–dead assay results in the PIC-RGD gels, we concluded that matrices with the 2 and 4 mg/mL PIC-RGD hydrogels are the most suitable candidates to fabricate the contraction assay. Both hydrogels show clear contraction responses which can be used to readily discriminate between the two cell types. As a consequence, we focused our studies further on the 2 and 4 mg/mL PIC-RGD hydrogel concentrations. In the following sections, we provide a more detailed picture on how cell behavior depends on the culture conditions and how we can exploit the differences between fibroblasts and myofibroblasts to further develop the contraction model.

### Biocompatibility of PIC-RGD Hydrogels

**Figure 4:**
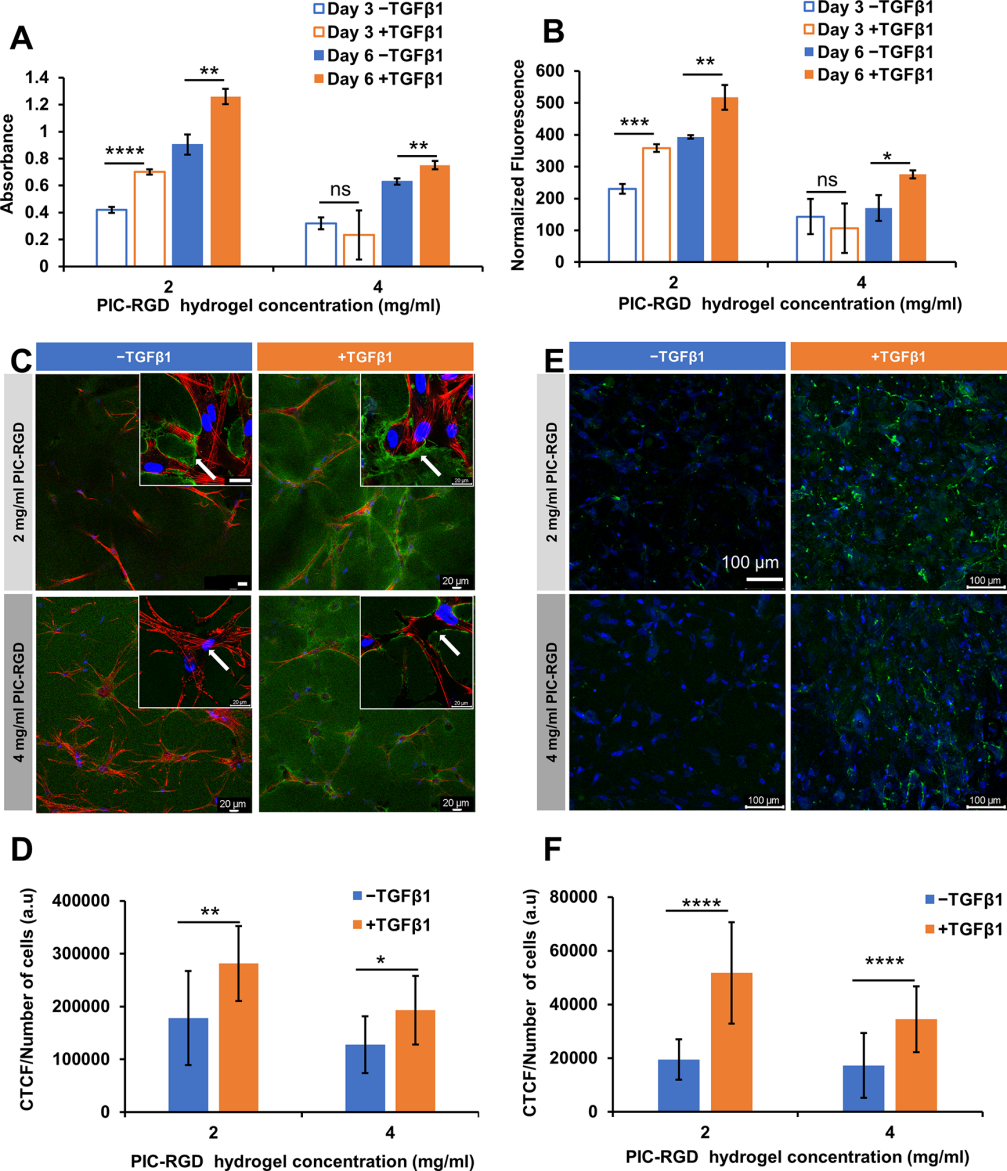
Cell viability, hydrogel staining, and total collagen production in PIC-RGD hydrogels seeded with fibroblasts in the absence or presence of TGFβ1. A) CCK8 assay results for cell viability at day 3 and day 6; *n* = 3. B) QuantiFluor total DNA quantification assay at day 3 and day 6; *n* = 3. C) Confocal image of the PIC-RGD cell cultures. Polymers were stained with DBCO-Cy3 (green), and the cytoskeleton was stained with Phalloidin Alexa Fluor 568 (red) and counterstained with DAPI for the nuclei (blue). D) Quantitative analysis of DBCO-Cy3 confocal images. The CTCF of the green channel was normalized to the number of cells; *n* = 12 images. E) Confocal fluorescence images of collagen stained with CNA35-OG488 (green) and counterstained with DAPI for the nuclei. F) Quantitative analysis of CNA35-OG488 confocal images; *n* = 22 images. Scale bars: panel C, 20 μm and panel E, 100 μm. Statistical analysis with an unpaired *t* test. *P*-values > 0.05 are considered not significant; significant differences: *, *P* ≤ 0.05; **, *P* ≤ 0.01; ***, *P* ≤ 0.001; ****, *P* ≤ 0.0001.

Besides viability, we analyzed mitochondrial activity through a CCK8 assay (Figure 4A) and cell densities through a QuantiFluor total DNA quantification (Figure 4B). The results show that in 2 mg/mL PIC-RGD, mitochondrial activity and total DNA content are higher for myofibroblasts than for fibroblasts and higher at day 6 than at day 3. The latter results suggest continued proliferation. In the more concentrated 4 mg/mL gel, the activation of the fibroblasts by TGFβ1 has a much smaller effect.

### Cell-Gel Interactions in PIC-RGD Hydrogels

In addition to the macroscopic contraction assay, we visualized matrix contraction at the cellular length scale by repeating the cell-culture experiment in fluorescently labeled (Cy3) PIC-RGD, which was introduced before. The Cy3 stained the pure hydrogel uniformly (Figure 1), but when cells are seeded with the hydrogel, the staining intensity of Cy3 was found to vary at different locations within the hydrogel, which we attribute to the local densification of the matrix as a result of cellular contraction (Figure 4C). We highlight that in the contracted hydrogel, the Cy3-stained fibers that are closer to the cells show increased fluorescence intensity. This effect was even stronger within the microenvironment of myofibroblasts than close to fibroblasts in both hydrogels (2 mg/mL, +/– TGFβ1: *P* = 0.0048; 4 mg/mL, +/– TGFβ1: *P* = 0.0134), which is in line with the observed macroscopic contraction. Furthermore, the increased fluorescence intensity of the gel near contracting cells was observed more in the softer gel where the polymer chains can be displaced easier (*P* = 0.0045) (Figure 4D). These results further support our finding that (macroscopic) gel contraction decreases with increasing concentration of the PIC-RGD gel.

### Total Collagen Production in PIC-RGD Gels

During fibrosis, the total amount of collagen often increases after deposition by myofibroblasts. Excreted collagen was visualized by staining with CNA35-OG488, which is a fluorescently labeled adhesion protein that specifically binds with collagen (Figure 4E). The quantification of green fluorescent signals by CTCF showed significantly higher signal in myofibroblasts compared to fibroblasts for both the 2 and 4 mg/mL PIC-RGD hydrogels, confirming increased collagen deposition by the myofibroblasts (Figure 4F).

### COL-1 and αSMA Production in PIC-RGD Gels

**Figure 5:**
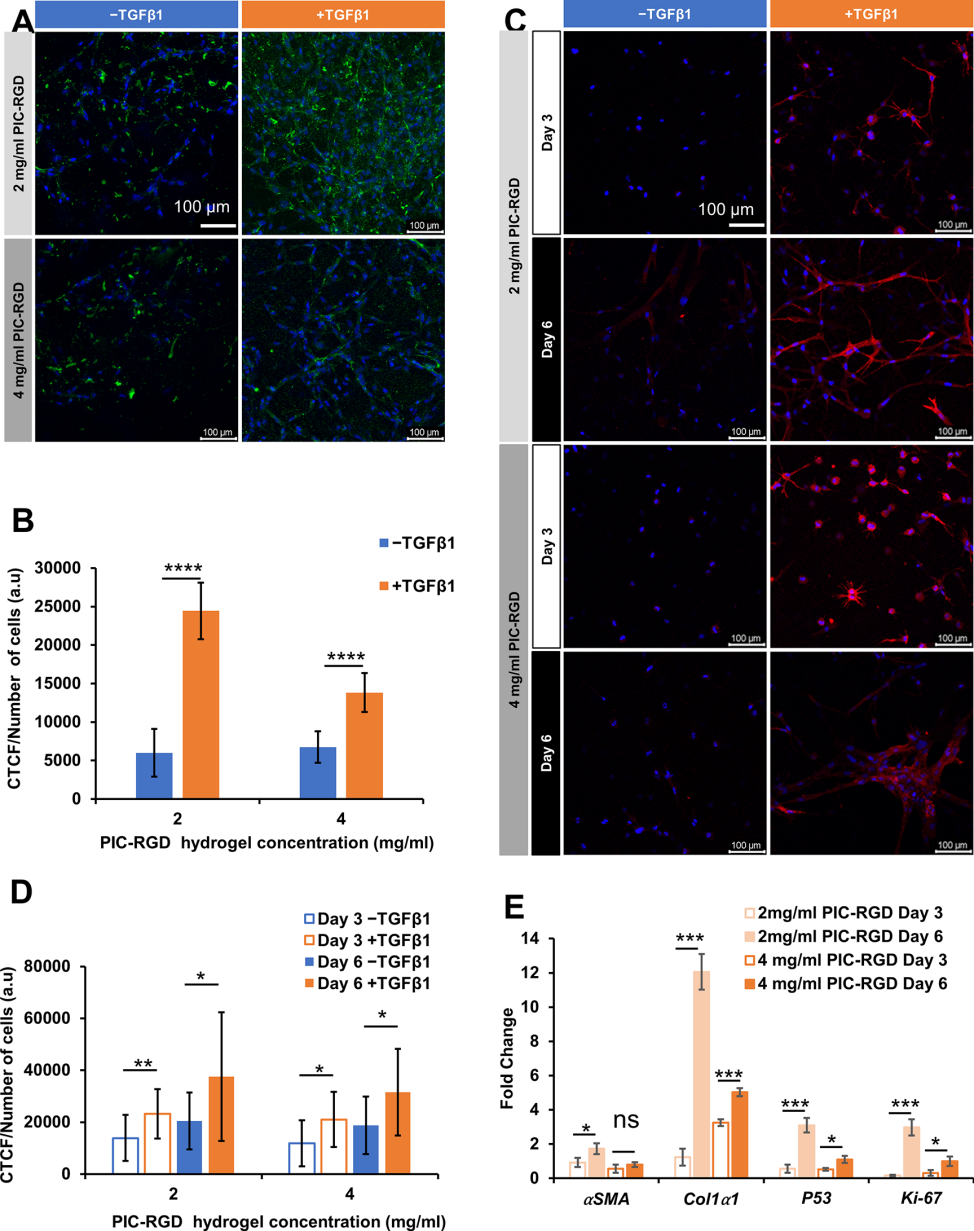
Immunostaining and gene expression analysis of fibroblasts cultured in the 2 and 4 mg/mL PIC-RGD hydrogels in the presence or absence of TGFβ1. A) Confocal fluorescence image of immunostaining with COL-1 (green) counterstained with DAPI for the nuclei (blue) at day 6. B) Quantitative image analysis of COL-1 production at day 6, normalized to the number of cells; *n* = 22. C) Confocal fluorescence image of immunostaining of α-SMA (red) counterstained with DAPI for the nuclei (blue) at days 3 and 6. D) Quantitative image analysis of α-SMA production, normalized to the number of cells; *n* = 16. E) mRNA gene-expression analysis of *α-SMA*, *Col1α1*, *P53*, and *Ki67* of fibroblasts and myofibroblasts in the 2 or 4 mg/mL PIC-RGD hydrogel at days 3 and 6. Scale bars in panels A and C: 100 μm. Statistical analysis with an unpaired *t* test. *P*-values > 0.05 are considered not significant; significant differences: *, *P* ≤ 0.05; **, *P* ≤ 0.01; ***, *P* ≤ 0.001; ****, *P* ≤ 0.0001.

The increased contraction in cultures with TGFβ1 is indicative of differentiation of the fibroblasts toward myofibroblasts. The transition was confirmed by immunostaining for COL-1 and αSMA. COL-1 staining showed increased expression in the presence of TGFβ1 for both the 2 and 4 mg/mL PIC-RGD hydrogels (Figures 5A and 5B). Further, αSMA immunostaining showed that at day 3 as well as at day 6 and for both hydrogel concentrations, the myofibroblasts expressed more αSMA than the fibroblasts (Figure 5C). In addition, quantitative analysis of the results showed a significantly increased number of αSMA-positive myofibroblasts at day 6 compared to day 3 (again for both PIC-RGD concentrations) for the cells cultured in the presence of TGFβ1 (Figure 5D). The results confirm that PIC-RGD hydrogels at day 6 are suitable as a fibrotic cell culture model.

To further validate fibroblast differentiation in PIC-RGD gels, we analyzed the expression of some genes related to fibrosis: *αSMA*, *Col1α1*, *p53*, and *Ki67*, where *αSMA* and *Col1α1* encode for αSMA and type I collagen, respectively, *p53* encodes for the transcription factor p53, which is strongly involved in wound healing^30^ (and cancer), and *Ki67* encodes for the proliferation marker Ki67. The expression levels are presented as the fold increase of expression in myofibroblasts, cultured in the presence of TGFβ1, with respect to fibroblasts (Figure 5E), normalized against the expression of household gene *GAPDH*. In the 2 mg/mL PIC-RGD hydrogels at day 6, gene expression in myofibroblasts was 1.7-, 12.0-, 3.0-, and 2.9-fold higher for *αSMA*, *Col1α1*, *p53*, and *Ki67*, respectively. On the other hand, in the 4 mg/mL PIC-RGD hydrogel at day 6, expression of *Col1α1* and *p53* was increased 5- and 1.1-fold, respectively, whereas a decrease in *αSMA* and *Ki67* expression in myofibroblasts was observed. Comparing the two sample sets at day 6, we found for all studied genes much higher expression levels in the 2 mg/mL matrix: 2.1-, 2.3-, 2.8-, and 2.9-fold for *αSMA*, *Col1α1*, *p53*, and *Ki67*, respectively.

### Nintedanib and Pirfenidone Reduce the Myofibroblast-Induced PIC-RGD Hydrogel Contraction, Proliferation, and Fibrotic Markers in PIC-RGD Hydrogels

To check the suitability of the PIC-RGD as an early stage fibrosis model, we studied the performance of two previously developed drugs, nintedanib and pirfenidone, in the 3D culture. Earlier studies in collagen-based contraction assays already showed the inhibitory effect of nintedanib and pirfenidone.^31−33^ In our experiments, we cultured HFFs in the presence of TGFβ1 to induce differentiation and added the drugs to the medium at day 6. At day 6, the drugs were administered with doses that have been used in the literature before.^32,34−36^ As a readout at day 7, we measured macroscopic contraction, cell proliferation, and expression of the myofibroblast marker αSMA at protein and gene levels.

**Figure 6:**
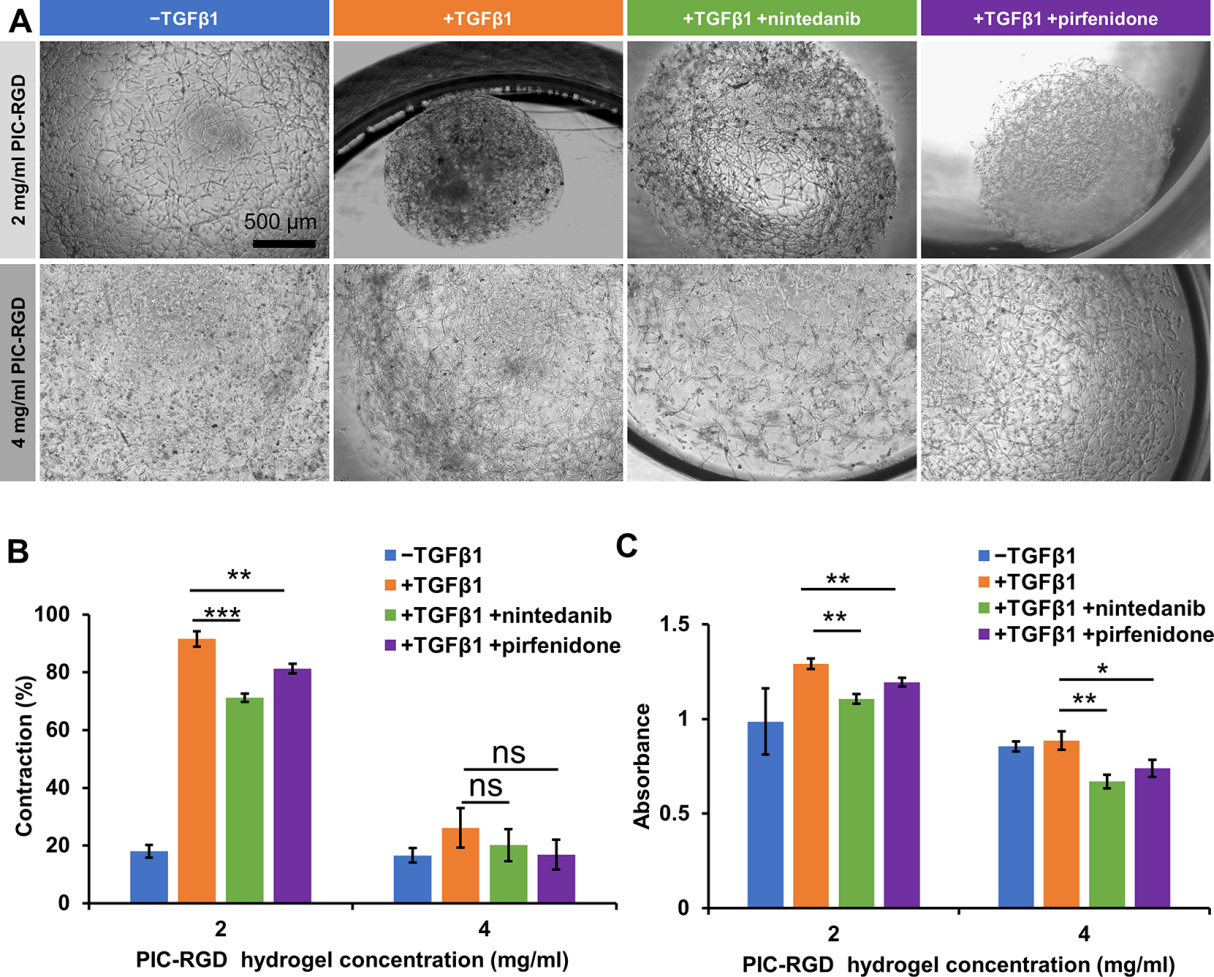
Effect of nintedanib and pirfenidone on contraction and cell viability after 24 h of treatment. A) Bright field microscopy images of hydrogels after drug treatment. B) Contraction of the hydrogels after drug treatment as a percentage of the gel size at day 0; *n* = 3. C) Cell viability assay by CCK8 analysis for cells after drug treatment; *n* = 3. Scale bar in panel A: 500 μm. Statistical analysis with an unpaired *t* test. *P*-values > 0.05 are considered not significant; significant differences: *, *P* ≤ 0.05; **, *P* ≤ 0.01; ***, *P* ≤ 0.001; ****, *P* ≤ 0.0001.

Bright field images after nintedanib and pirfenidone exposure showed that contraction decreased significantly in the 2 mg/mL hydrogel (fold change by 0.77 and 0.88; *P* = 0.003 and *P* = 0.004), respectively, compared to no treatment, albeit the levels do not return to those of fibroblasts cultured in the absence of TGFβ1 (Figure 6A,B). In contrast, in the stiffer 4 mg/mL hydrogels where contraction under the influence of TGFβ1 is less prominent, the decrease due to nintedanib or pirfenidone treatment was statistically not significant.

**Figure 7:**
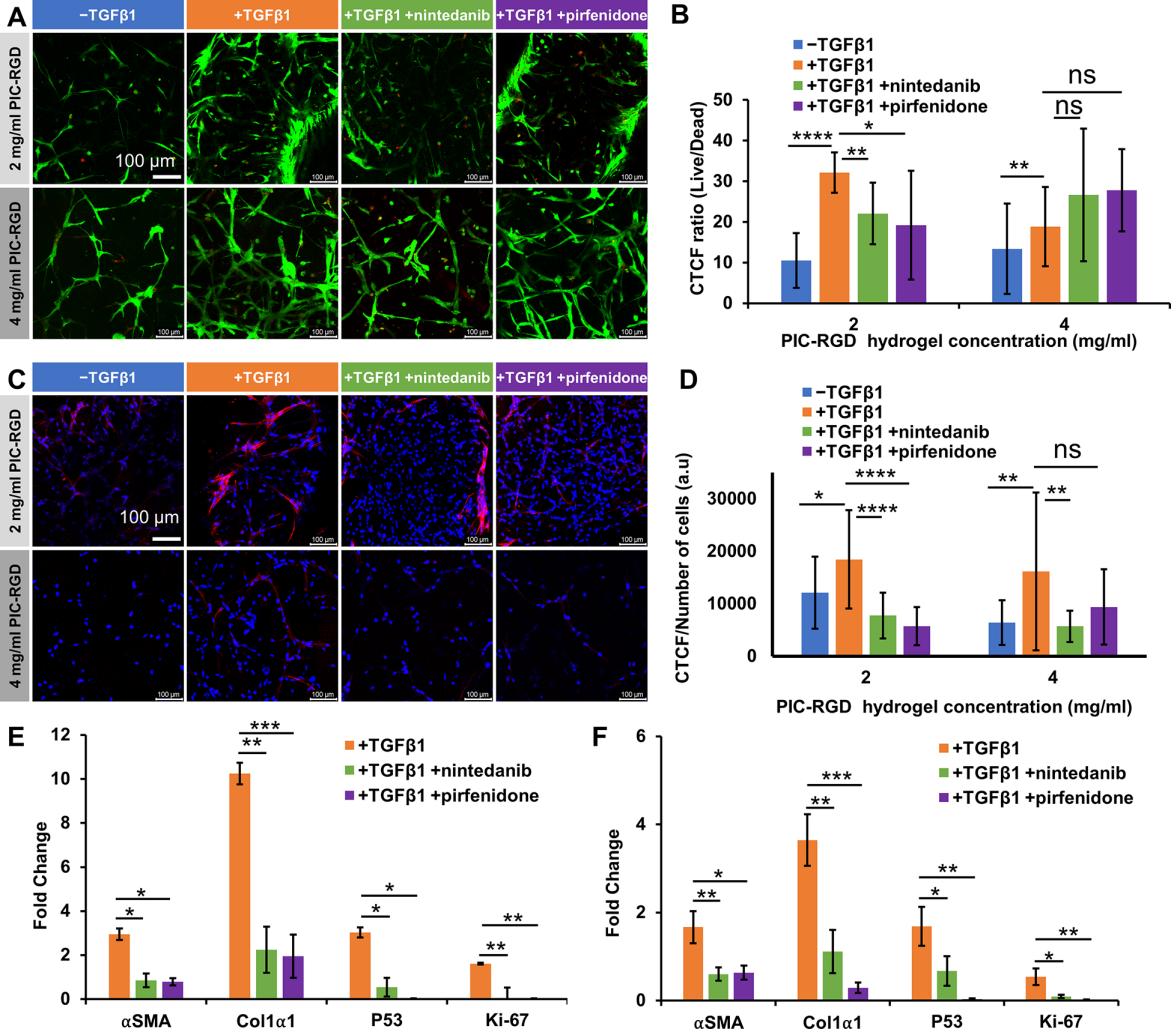
Effect of nintedanib and pirfenidone on protein and gene expression after 24 h of treatment. A) Confocal images of the live–dead assays at day 7. Living cells were stained with Calcein-AM (green); dead cells were stained with TOTO-3 (red). B) Quantitative image analysis of live–dead assay results and CTCF presented as the ratio of live to dead cells; *n* = 8. C) Confocal fluorescence image of immunostaining of α-SMA (red) counterstained with DAPI for the nuclei (blue) in both hydrogels. D) Quantitative image analysis of α-SMA production, normalized to the number of cells; *n* = 20. E,F) mRNA gene-expression analysis of *α-SMA*, *Col1α1*, *P53*, and *Ki67* for treated and nontreated samples in the 2 mg/mL PIC-RGD hydrogel (E) and in the 4 mg/mL PIC-RGD hydrogel (F). Scale bars in panels A and C: 100 μm. Statistical analysis with an unpaired *t* test. *P*-values > 0.05 are considered not significant; significant differences: *, *P* ≤ 0.05; **, *P* ≤ 0.01; ***, *P* ≤ 0.001; ****, *P* ≤ 0.0001.

In line with previous studies showing that nintedanib and pirfenidone reduce TGFβ1-induced proliferation of human fibroblasts,^7,10^ we observed that in our PIC-RGD hydrogel fibrosis model both nintedanib and pirfenidone significantly reduced myofibroblast proliferation (Figure 6C). This reduction in the case of 2 mg/mL was 0.85- and 0.92-fold, whereas in 4 mg/mL, it was 0.75- and 0.83-fold after nintedanib and pirfenidone treatment, respectively, compared to the nontreated group. The reduced proliferation is stronger for nintedanib and similar in extent in the 2 and 4 mg/mL hydrogels. The live–dead assay showed that most of the cells were still viable after nintedanib or pirfenidone treatment in the PIC-RGD hydrogel (Figure 7A). The quantitative analysis of the live–dead confocal images showed the CTCF ratio of live to dead cells was higher than one for all groups (Figure 7B). The result further confirms the viability of the cells after drug treatment. Further, in the case of the 2 mg/mL PIC-RGD hydrogel, the ratio of live to dead cells was reduced in the nintedanib (*p* = 0.0072) and pirfenidone (*p* = 0.0221) groups compared to the TGFβ1-treated groups. This result is in line with the proliferation assay at of 2 mg/mL. In the 4 mg/mL hydrogels, no significant differences were observed in the ratio of live to dead cells for the drug-treated groups compared to the TGFβ1-treated groups.

To confirm the reduction of the fibrotic character of the culture as indicated by the contraction and proliferation assays, we determined the production of the myofibroblast marker αSMA by immunostaining and studied gene expression of *αSMA*, *Col1α1*, *p53*, and *Ki67*. Fluorescence microscopy of the immunostaining experiments (Figure 7C) indicated a reduction of αSMA expression as a result of drug treatment in all cultures. Quantification of the αSMA confocal images showed a decrease of αSMA after treatment with nintedanib (0.42-fold, *P* = 0.0001) or pirfenidone (0.31-fold, *P* = 0.0001) in the 2 mg/mL PIC-RGD hydrogel, compared to the nontreated myofibroblasts (Figure 7D). For the 4 mg/mL hydrogel, the decrease in αSMA was seen only in the nintedanib group (0.35-fold, *P* = 0.0041) but not in pirfenidone-treated samples.

The PCR experiments further show that the expression of all four tested genes is suppressed by the antifibrotic drugs. The reduced *αSMA* expression is in line with the immunostaining results, and associated with that, one could also expect a decrease in *Col1α1*. We note that *p53* and *Ki67* show an even stronger reduction as a result of treatment with pirfenidone as compared to nintedanib (Figures 7E and 7F).

## Discussion

To develop *in vitro* contraction and fibrosis models providing a suitable microenvironment is critical; the matrix drives cell behavior and morphologies, illustrated by normal fibroblasts that show a more myofibroblast-like phenotype when grown on ECM deposited by fibrotic fibroblasts.^37^ This interaction should be considered across the biomedical field, including in the setup of drug screening assays. In this work, we demonstrated that PIC-RGD hydrogels can be effectively used to screen putative fibrotic drugs on their efficacy to inhibit contraction, which constitutes a very simple readout that is compatible with high-throughput processes.

### PIC-RGD Gels as a Small Sample Contraction-Based Fibrosis Model

As the mechanical properties are important for contraction, we first screened hydrogels with different polymer concentrations (1–8 mg/mL) with different stiffnesses, for discrimination in contraction by fibroblasts and myofibroblasts, modeling fibrotic and healthy tissue, respectively. We found that at 1 mg/mL, the gels are too soft and fragile; they contract and even break during the 6 days in culture. At 8 mg/mL, the gels hardly contracted at all. We continued with the 2 and 4 mg/mL gels, which both contracted more strongly for the myofibroblasts than for the fibroblasts, both macroscopically and microscopically through hydrogel labeling.^38,39^ The contractility of PIC hydrogels seems strongly related to their stiffness and possibly less to the strain stiffening properties of PIC gels, which are expected to increase the stiffness upon cellular contraction.^19^ We emphasize that the different concentration gels will also differ in porosity and the density of cell adhesion sites. For further optimization, these parameters can be tailored independently in PIC gels, similar to many other synthetic hydrogels.

Earlier work showed that the mechanical properties of the fibroblast microenvironment critically determine cell migration, spreading, and differentiation.^40,41^ Additionally, enhanced cell spreading promotes increased levels of F-actin and αSMA.^42^ In our work, we find that in the softer (2 mg/mL) gels (with the lower RGD concentration), the cells spread more easily, which corresponds to earlier work.^43^ Furthermore, myofibroblasts show a more spread-out morphology than fibroblasts in any of the gels. Here, for a skin fibrosis model, we find for our human foreskin fibroblasts an optimal PIC-RGD concentration of 2–4 mg/mL; for other fibroblasts, these conditions may require reoptimization.

In addition, we studied other cell characteristics that are associated with fibrosis. Cellular proliferation of myofibroblasts was higher than for fibroblasts, which corresponds to previous work that demonstrated that TGFβ1 increases the proliferation of human fibroblasts.^44^ Myofibroblasts are known to produce both αSMA and excessive collagen type I, which contribute to fibrosis and scar formation.^45^ Indeed, in the TGFβ1-treated fibroblasts, we observed an increased production of total collagen, αSMA, and collagen type 1 compared to nontreated fibroblasts, which is in line with the literature.^46^ We note that tracking cellular collagen excretion obviously is easier in noncollagenous matrices than in the “default” collagen-based contraction assays or in other assays from biological origins. Increased gene expression of *αSMA* and *Col1α1* in myofibroblasts supports the increased protein production.^1,45^

In addition, the myofibroblasts show increased expression of *p53* and *Ki67*. During renal fibrosis, TGFβ1 activates reactive oxygen species-dependent pathways, thereby inducing gene expression of p53 and EGFR.^47,48^ The former, as a coactivator of TGFβ1, controls apoptosis, cell growth, and stress responses and promotes fibrosis.^48,49^ Targeting *p53* gene expression is a valid strategy to mitigate fibrosis.^49^ Easy detection of (increased) *p53* expression in the small scale samples is very attractive in developing drugs against fibrosis and other diseases, including cancer. Ki67 is a marker for cell proliferation, and the increased *Ki67* expression from day 3 to day 6 suggests that the myofibroblasts continue to proliferate. We note that all studied gene expressions increased more in the 2 mg/mL PIC-RGD hydrogel than in the 4 mg/mL gel, which indicated that this softer matrix provides a more suitable microenvironment to discriminate between healthy and diseased tissues and to observe the effects of potential drugs. Since the expression continued to increase from day 3 to day 6, then the day 6 time point was selected to screen the effects of drugs against fibrosis and scarring.

### Model Validation Using Existing Antifibrosis Drugs

Nintedanib and pirfenidone are the only FDA-approved drugs to treat pulmonary fibrosis.^50^ Additionally, nintedanib showed its antifibrotic effect in a mouse model of systemic sclerosis,^34^ and pirfenidone showed its therapeutic use in scleroderma patients.^51^ Moreover, nintedanib is known to inhibit TGFβ1-induced myofibroblast proliferation and gene expression,^32^ and consequently, we may expect that both drugs will affect particularly the myofibroblast cultures in cell proliferation and expression levels of all studied genes, as well as the production of αSMA and collagen.

Thus, to validate our PIC-RGD model, we tested the effects of nintedanib and pirfenidone on fibrosis and scarring parameters. The decrease in matrix contraction and myofibroblast proliferation after treatment with nintedanib and pirfenidone proves its efficacy and its suitability for screening of antifibrotic drugs and molecular analyses. We note that while the 4 mg/mL PIC-RGD hydrogel did not show significant effects in terms of contraction in the drug-treated myofibroblast cultures, the efficacy of the drugs did show in the cell viability and gene expression experiments.

### Comparison to Collagen-Based Assays

Currently, the gold standard in contraction assays is not based on a synthetic polymer but on collagen. Consequently, we added a collagen-based positive control to our studies, with 0.5 and 1 mg/mL collagen, which was applied similarly to the PIC-RGD gels, with similar volumes (10 μL) and cell densities. We note that this volume is much smaller than what is commonly used in collagen-based assays. As a result, we observe a different behavior. Already within 1 day, the collagen gels, both with fibroblasts and with myofibroblasts, have completely contracted to <5% of their original size. The contraction reduced cell viability leading to a high mortality rates at days 3 and 6. Our observations are in line with earlier work^52^ that showed that fibroblasts encapsulated in 3D contractile collagen gels are more susceptible to apoptosis. Recently, another study suggested that hydrogels with lower collagen concentrations (0.5 mg/mL) induce higher contraction levels and lower cell viability compared to more concentrated gels (2 mg/mL).^53^

The benefit of PIC-RGD gels, however, is not that the results of larger collagen assays can be reproduced. Besides nice practicalities such as the absence of batch-to-batch variations and low autofluorescence, there are many major advantages, for instance the following: (*i*) The small (10 μL) scale, which is very attractive for screening purposes but still allows for thorough (quantitative) staining and RNA expression analysis. (*ii*) As a thermoresponsive gel, it is incredibly easy to extract the cells as well as the secretome for further downstream analysis; both will not be contaminated with components that originate from the matrix, which is a major disadvantage of Matrigel and other animal-derived materials.^54^ (*iii*) Maybe the most interesting advantage is that because of its synthetic nature, the PIC-RGD matrix can be readily tailored not only in physical properties but also in biochemical cue (proteins, growth factors, etc.) readily conjugated to the gel, allowing one to really optimize the material toward a desired application.

## Conclusion

In summary, we found that the PIC-RGD hydrogel is highly suitable as an *in vitro* contraction and fibrosis platform to monitor the efficacy of various drugs and chemicals on fibrosis, scarring through contraction, and molecular and cellular analyses. For proof-of-concept purposes, only the polymer concentration was optimized, but the model is easily finetuned further. The 2 mg/mL PIC-RGD hydrogels showed a clear discrimination in contraction properties and cell spreading of fibroblasts and myofibroblasts. Drug screening results showed that both FDA-approved antifibrotic drugs potently inhibited myofibroblast contraction in this hydrogel. Moreover, the PIC-based gels allow for subsequent RT-PCR and immunofluorescence staining, which demonstrated that fibrotic gene and protein expression was affected by the drugs. The small scale, easy handling, and high versatility of the PIC gel make this material an attractive candidate for high-throughput screening of putative drugs against fibrosis and scarring and potentially toward other diseases.

## Supporting Information Available

Additional experimental details, including mechanical data, cell culture, and contraction data (PDF)
