# Supplementary material for: Novel Synthetic Polymer-Based 3D Contraction Assay: A Versatile Preclinical Research Platform for Fibrosis
Source: ACS Appl Mater Interfaces. 2022 Apr 25;14(17):19212–25. doi: 10.1021/acsami.2c02549 (PMC9073832; doi:10.1021/acsami.2c02549)
Supplement: Supplementary file 1 — am2c02549_si_001.pdf [file am2c02549_si_001.pdf]

Supporting information for

A novel synthetic polymer-based 3D contraction assay: a versatile preclinical research platform for fibrosis

Jyoti Kumari[a,b], *Frank A. D. T. G. Wagener*[b,]* *and Paul H. J. Kouwer*[a,]*

[a] Institute for Molecules and Materials, Radboud University, Heyendaalseweg 135, 6525 AJ, Nijmegen, The Netherlands

[b] Department of Dentistry - Orthodontics and Craniofacial Biology, Radboud University Medical Centre, Nijmegen, The Netherlands

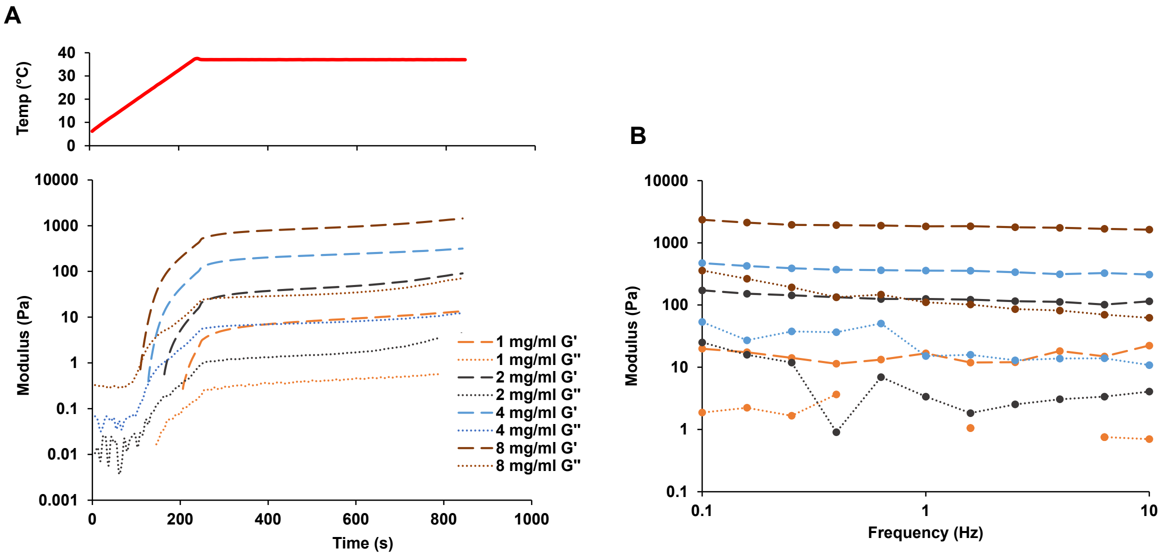

Figure S1: Storage modulus *G′* and loss modulus *G″* of different concentrations PIC-RGD hydrogels: A) Time trace of heating experiment (ω = 1 Hz); B) Frequency sweep (γ = 2 %, T = 37 °C).

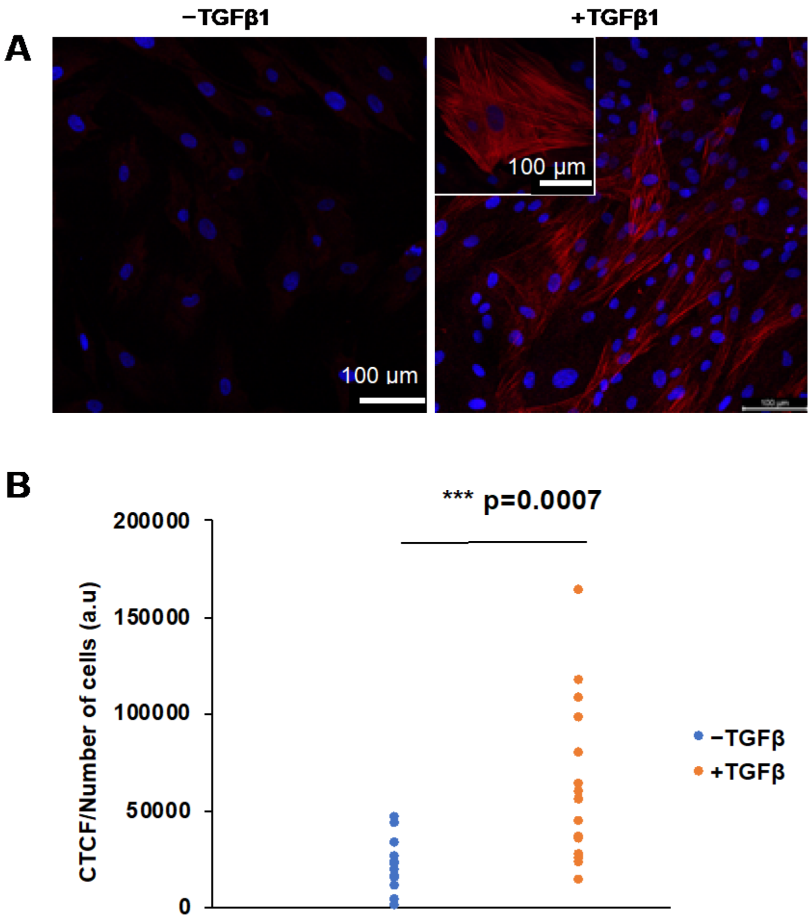

Figure S2: Immunostaining of fibroblasts of αSMA (red), counterstained with DAPI for the nucleus (blue) cultured in 2D on tissue culture plates in absence and presence of TGFβ1**.** B) Quantitative analysis of confocal images, CTCF of the red channel was calculated and normalized to the cell number; *n*=16 images. Scale bar (A): 100 μm. Statistics: Unpaired t-test was performed to determine the P-value.

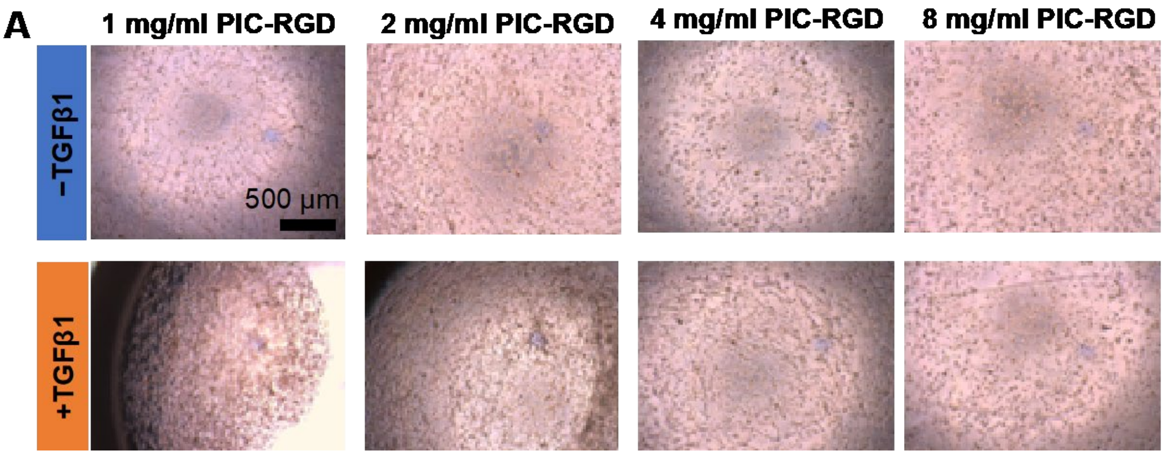

Figure S3: Bright field images showing contraction in 1,2, 4 and 8 mg/ml PIC-RGD hydrogel seeded with fibroblasts in the absence or presence of TGFβ1 at day 3. Scale bar (A): 500 µm.

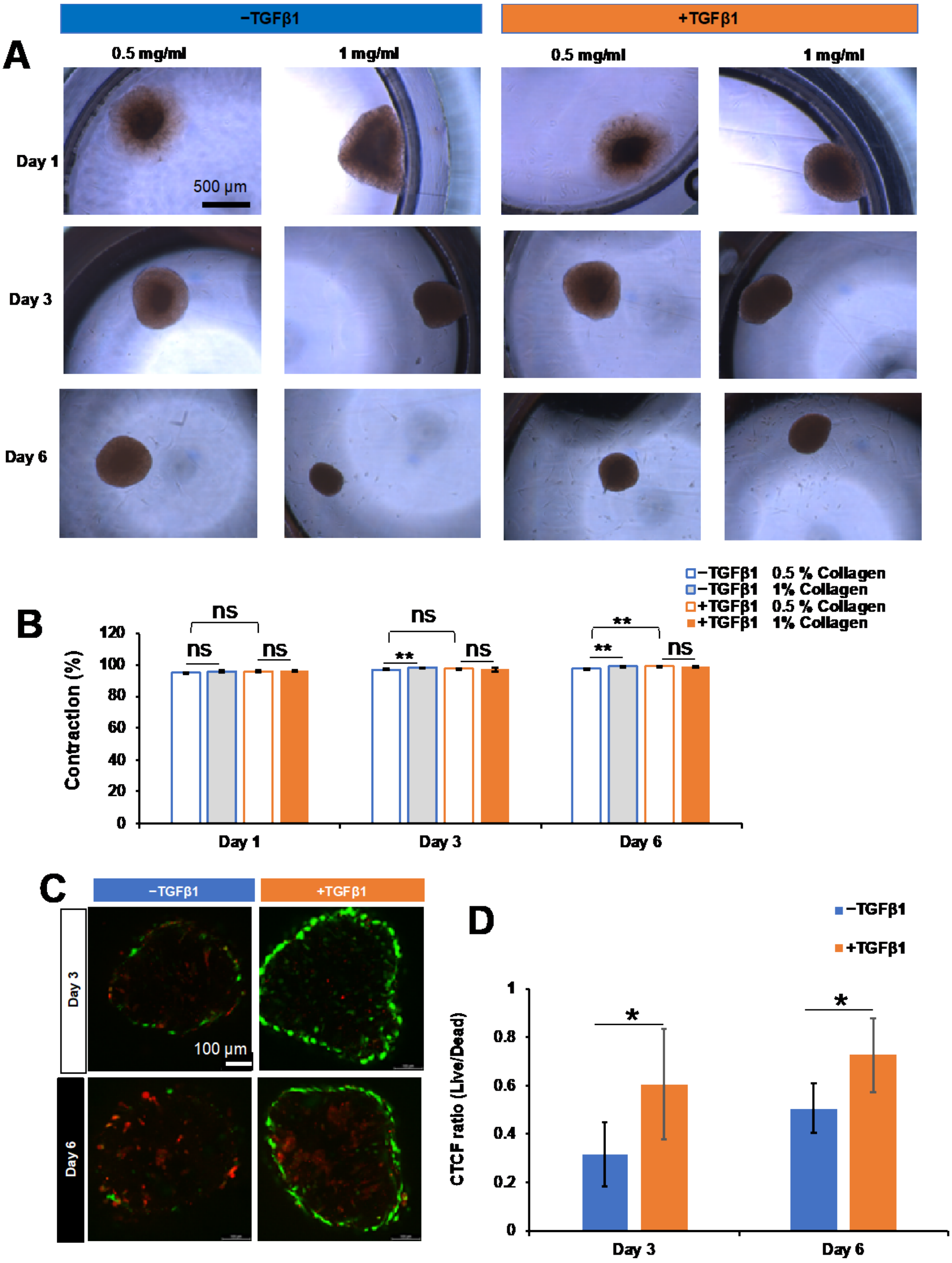

Figure S4: Contraction analysis and lived dead staining in collagen hydrogels. A) Bright field images showing contraction in 0.5 and 1 mg/ml collagen hydrogels seeded with fibroblasts in the absence or presence of TGFβ1 at day 1, 3 and 6. B) Percentage contraction measurement of the hydrogel at days day 1, 3 and 6. *n*=3 independent samples. C) Confocal images of live-dead assays at day 3 and day 6. Living cells (green) and dead cells (red) were stained with Calcein-AM and Toto-3, respectively. D) Quantitative analysis of live dead confocal images, CTCF of green and red channel was calculated and presented as ratio. n=8. Scale bar (A): 500 µm, (C): 100 µm. Unpaired t-test was performed to determine the *P*-value. *, P ≤ 0.05, **, P ≤ 0.01, ns, not significant.
